# Structural characterization of green fluorescent protein in the I-state

**DOI:** 10.1038/s41598-024-73696-y

**Published:** 2024-10-01

**Authors:** Ryota Takeda, Erika Tsutsumi, Kei Okatsu, Shuya Fukai, Kazuki Takeda

**Affiliations:** Department of Chemistry, Graduate School of Science, Kyoto University, Sakyo-ku, Kyoto, 606-8502 Japan

**Keywords:** Fluorescent protein, Hydrogen bond, Intermediate, Non-covalent interaction, QM/MM, X-ray crystallography, Biophysics, Structural biology

## Abstract

Green fluorescent protein (GFP) is widely utilized as a fluorescent tag in biochemical fields. Whereas the intermediate (I) state has been proposed in the photoreaction cycle in addition to the A and B states, until now the structure of I has only been estimated by computational studies. In this paper, we report the crystal structures of the I stabilizing variants of GFP at high resolutions where respective atoms can be observed separately. Comparison with the structures in the other states highlights the structural feature of the I state. The side chain of one of the substituted residues, Val203, adopts the *gauche-* conformation observed for Thr203 in the A state, which is different from the B state. On the other hand, His148 interacts with the chromophore by ordinary hydrogen bonding with a distance of 2.85 Å, while the weaker interaction by longer distances is observed in the A state. Therefore, it was indicated that it is possible to distinguish three states A, B and I by the two hydrogen bond distances Oγ-Thr203···Oη-chromophore and Nδ1-His148···Oη-chromophore. We discuss the characteristics of the I intermediate of wild-type GFP on the bases of the structure estimated from the variant structures by quantum chemical calculations.

## Introduction

**Fig. 1:**
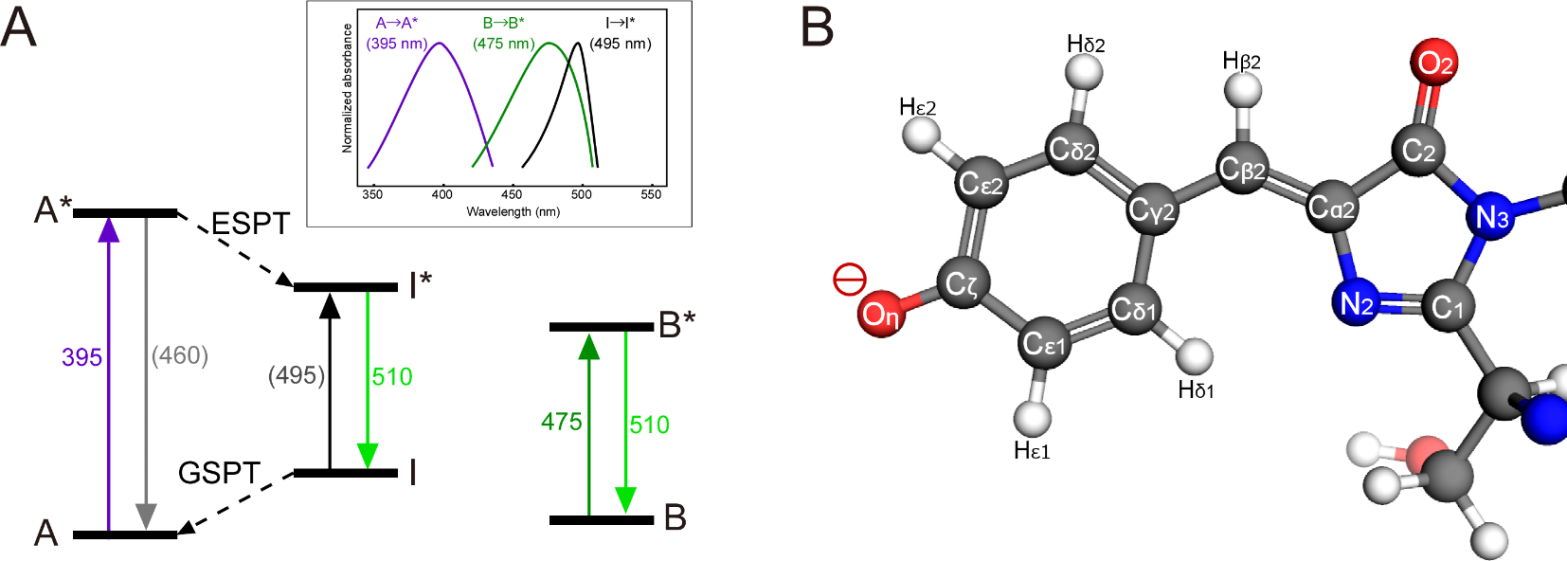
The A, B and I states of GFP. (**A**) A schematic representation of the energy diagram of wild-type GFP. The values indicate approximate wavelengths of absorption and emission in units of nm. The inset indicates schematic absorption spectra for the three states. (**B**) The structure of the anionic chromophore in the I state. Names of atoms in the chromophore are indicated.

Green fluorescent protein (GFP), discovered from *Aequorea victoria*, emits green light after absorption of blue light^1^. The emission requires no extrinsic cofactors and is therefore widely utilized as a fluorescent tag or sensor in biochemical fields^2–4^. GFP has a β-barrel fold, and the chromophore is formed at the center from the three amino acid residues Ser65, Tyr66 and Gly67^5,6^. The suppression of the fluctuations of the chromophore by surrounding amino acid residues is crucial for its emission capacity^7,8^. The chromophore of wild-type GFP in the ground state exhibits polymorphism in the chemical structure^9^. The protonation state of the chromophore differs between the two states, A and B. The phenolic group in the chromophore is protonated and neutral in the A state, while it is deprotonated and negatively charged in the B state. The A state is predominant in wild-type GFP^10,11^. Spectroscopic measurements of the photoreaction of the A-type chromophore revealed the presence of the excited state proton transfer (ESPT) reaction (Fig. 1A), and a photoreaction model was proposed that includes the intermediate (I) state (Fig. 1B) in addition to the A and B states^10^. After photo-excitation of the A-type chromophore by purple light of 395 nm (A → A*), a change in electronic state leads to a structural change with deprotonation by an excited state proton transfer reaction (A* → I*), and emission of green light of 510 nm occurs via the I state (I* → I)^10^. The I state then returns to the A state (I → A) by a ground state proton transfer (GSPT) reaction. The ESPT and GSPT reactions are proton relays in the chromophore-water-Ser205-Glu222 hydrogen bond network^11^, and the final proton acceptor of the ESPT reaction is Glu222^12^. Although it rarely occurs under normal circumstances, the I → I* transition due to the transient I state is 495 nm (Fig. 1A), which is a considerably longer wavelength than the B → B* transition of 475 nm where the chromophore is similarly deprotonated^10,13^.

The atomic structures of GFP in the I state have only been constructed by computational studies^14,15^. However, there were differences between these studies in the structure of the residues around the chromophore, and the details of the structure and interactions of the chromophore and its surrounding residues in the I state were not clear. Substitutions of residues around the chromophore can yield variant proteins that are stabilized in a single specific state. The A state is stabilized in the T203V and T203I variants of GFP, while the B state is stabilized in the E222Q variant^16–18^. Furthermore, the T203V/E222Q variant, which combines these substitutions, has spectroscopic features of the I state^19^, and indeed has been utilized as an analogue of the I state^12,18^.

In this study, the structural features of GFP in the I state are investigated by determining the crystal structures of the I-variants and comparing them with the structures of variants in the other states. In addition, the structure of the I intermediate of wild-type GFP is proposed based on these variant structures, and the proton transfer mechanism in GFP is discussed.

## Results

### Preparation and characterization of variants

**Fig. 2:**
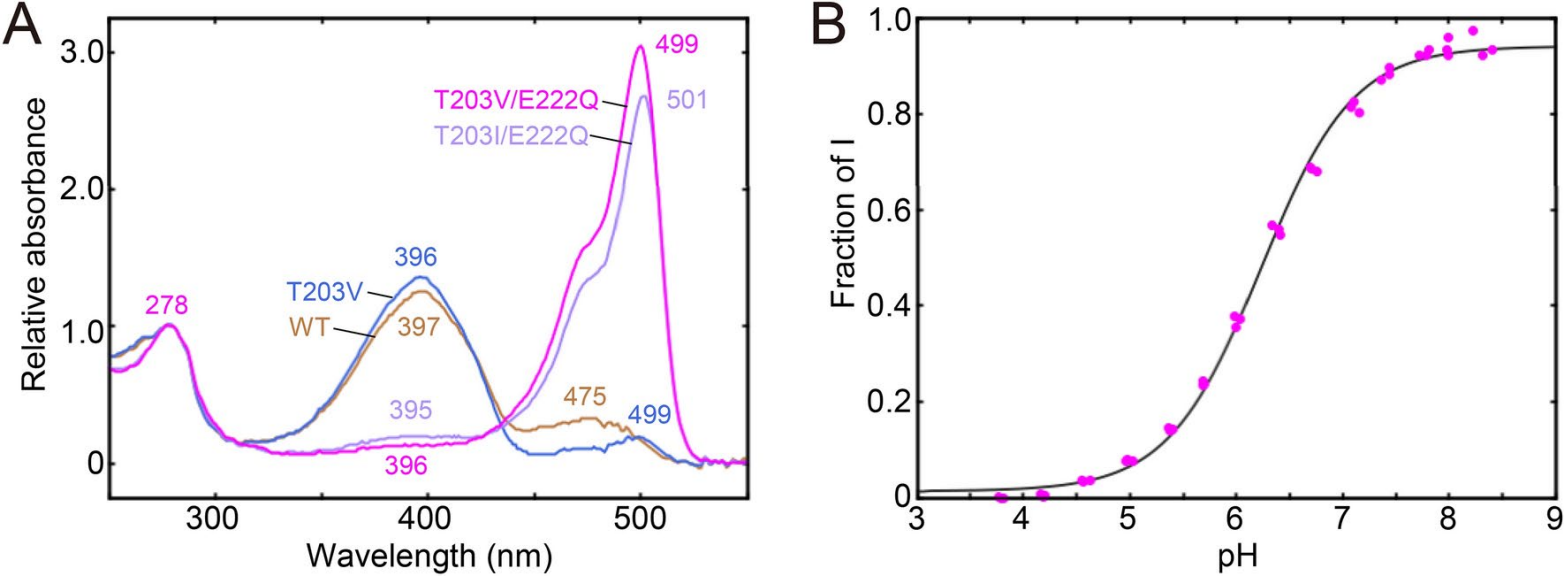
The pH dependence of the UV–vis absorption spectra. (**A**) UV–Vis absorption spectra of variant GFPs at pH 7.4. Magenta, light purple, blue and beige lines are for T203V/E222Q, T203I/E222Q, T203V and wild-type GFP, respectively. The absorbances at 278 nm were scaled to be 1.0 for the plot, while the original absorbances were ~ 0.1 for all variants. The values in the figure indicate peak wavelengths of the respective variants in units of nm. (**B**) The pH titration. The fraction of the I state for the T203V/E222Q variant is plotted against the pH values as magenta closed circles. The overlayed curve is determined by least square fitting.

In this study, we focused on structures of the T203V/E222Q and T203I/E222Q variant which are predominantly in the I state at physiological pH (with a small contribution from the A state)^19^, the T203V variant which is predominantly in the A state (with a negligible contribution from the I state)^16–18^, and wild-type GFP which is mainly in the A state (with a small contribution from the B state)^10,11^.To achieve high yields, the cycle3 mutation, which elicits no apparent changes in spectral and structural features, was additionally introduced to the variants^20–22^. The yield of the purified sample was 20 mg per liter of culture for the T203V/E222Q variant, which is comparable to the yield of ~ 30 mg per 1 L of culture for the S65T variant^23^. The UV–Vis absorption spectrum of the variant at pH 7.4 has two peaks in the visible region in addition to a peak at ~ 280 nm in the UV region (Fig. 2A). Whereas the absorption peak at 396 nm is due to the A state, the peak at 499 nm is due to the I state^13^. The wavelengths of the visible peaks are almost the same as the previously reported values^19^, despite the introduction of the cycle3 mutation. Isoleucine is very similar to valine in structure and properties, and it has been reported that the A state is selectively stabilized both in the T203V and T203I variants^17^. Therefore, to investigate the stabilization of the I state by mutation in detail, we additionally constructed the T203I/E222Q variant with the cycle3 mutation resembling the T203V/E222Q variant. The yield of purified sample of the T203I/E222Q variant was 4 mg per liter of culture, one-fifth less than that of the T203V/E222Q variant. The peak wavelengths of the UV–Vis absorption spectrum at pH 7.4 are almost identical to those of the T203V/E222Q variant, indicating that the I state is also stabilized in the T203I/E222Q variant (Fig. 2A). The pH dependence of the absorption spectra was measured for the T203V/E222Q variant, which exhibits the higher yield of the two variants (Fig. S1). The fraction of the I state increases as the pH increases (Fig. 2B). The major fraction of T203V/E222Q (> 90%) is in the I state under basic conditions above pH 8.0, while it is in the A state under acidic conditions below pH 5.0. The pK_a_ value of the T203V/E222Q variant is determined to be 6 ± 1 from the pH titration curve.

### Crystallographic structure analysis

**Fig. 3:**
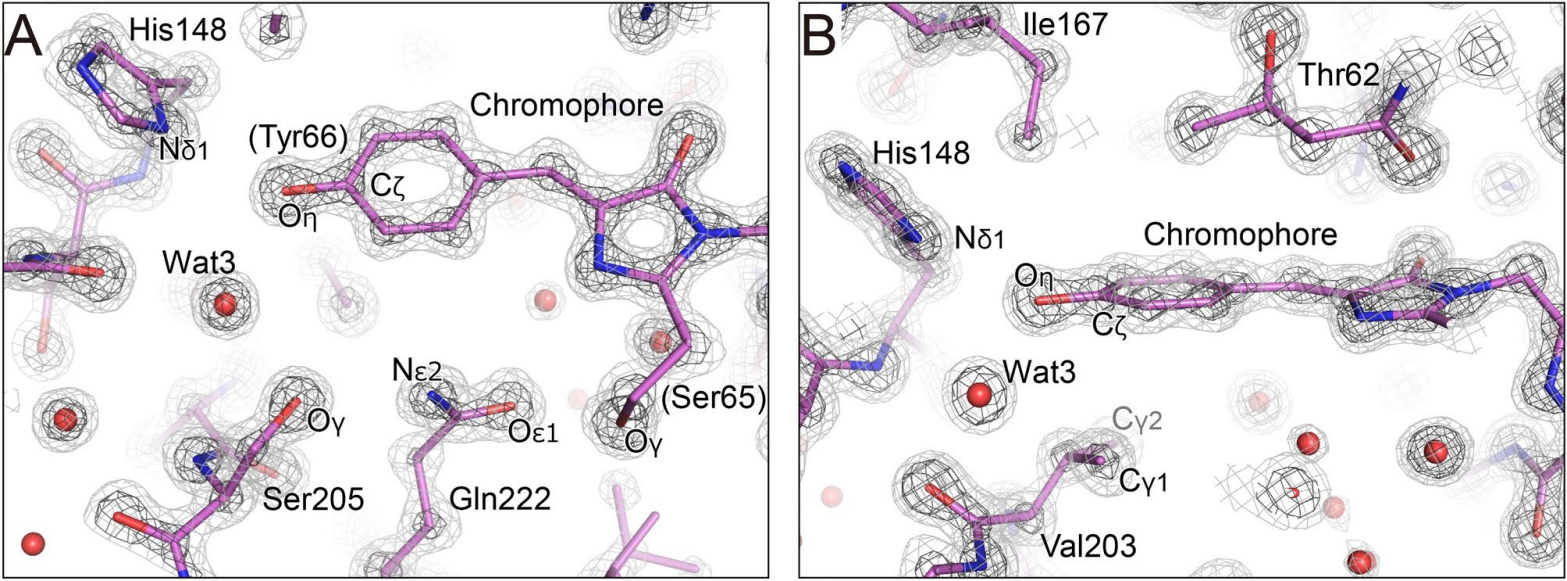
Crystal structures of GFP in the I state. (**A**) The 2m*F*_obs_ – D*F*_calc_ map around the chromophore of the T203V/E222Q variant at pH 8.5 is shown as light gray and gray meshes at the 1.5σ and 3σ levels. (**B**) The side view of (**A**).

The crystal structure of the T203V/E222Q variant at pH 8.5 was determined at a high resolution of 1.2 Å where the respective non-hydrogen atoms can be observed separately (Fig. 3). The diffraction precision index (DPI) for the average coordinates error was estimated to be 0.07 Å. Crystallographic and refinement statistics are listed in Table S1. The quality of the electron density map was sufficient to distinguish between nitrogen and oxygen atoms and between carbon and nitrogen atoms. Thus, the conformations of residues such as His148, Val203 and Gln222 around the chromophore could be accurately determined without ambiguity. The Nδ1 atom of His148 forms a hydrogen bond with the Oη atom of the chromophore. The Nε2 and Oε1 atoms of Gln222 make hydrogen bonds with the Oγ atom of Ser205 and the Oγ atom of Ser65 in the chromophore, respectively (Fig. 3A). The side chain of the substituted residue Val203 takes the *gauche-* conformation (Fig. 3B), similar to Thr203 in wild-type GFP^6,11^. The Cγ1 atom of Val203 has a distance of 3.45 Å from the Cζ atom of the chromophore, implying the presence of a van der Waals interaction between the two atoms (Fig. 3B).

The crystal structure of the T203I/E222Q variant was determined at 1.48 Å resolution (Fig. S2A,B). The distance between Nδ1 of His148 and Oη of the chromophore is 2.85 Å (Fig. S2C). Ile203 take a conformation similar to Val203 in the T203V/E222Q variant, and the interaction manner with the chromophore is also similar with the T203V/E222Q variant (Fig. S2C,D). Gln222 also take the same conformation as that in the variant.

**Fig. 4:**
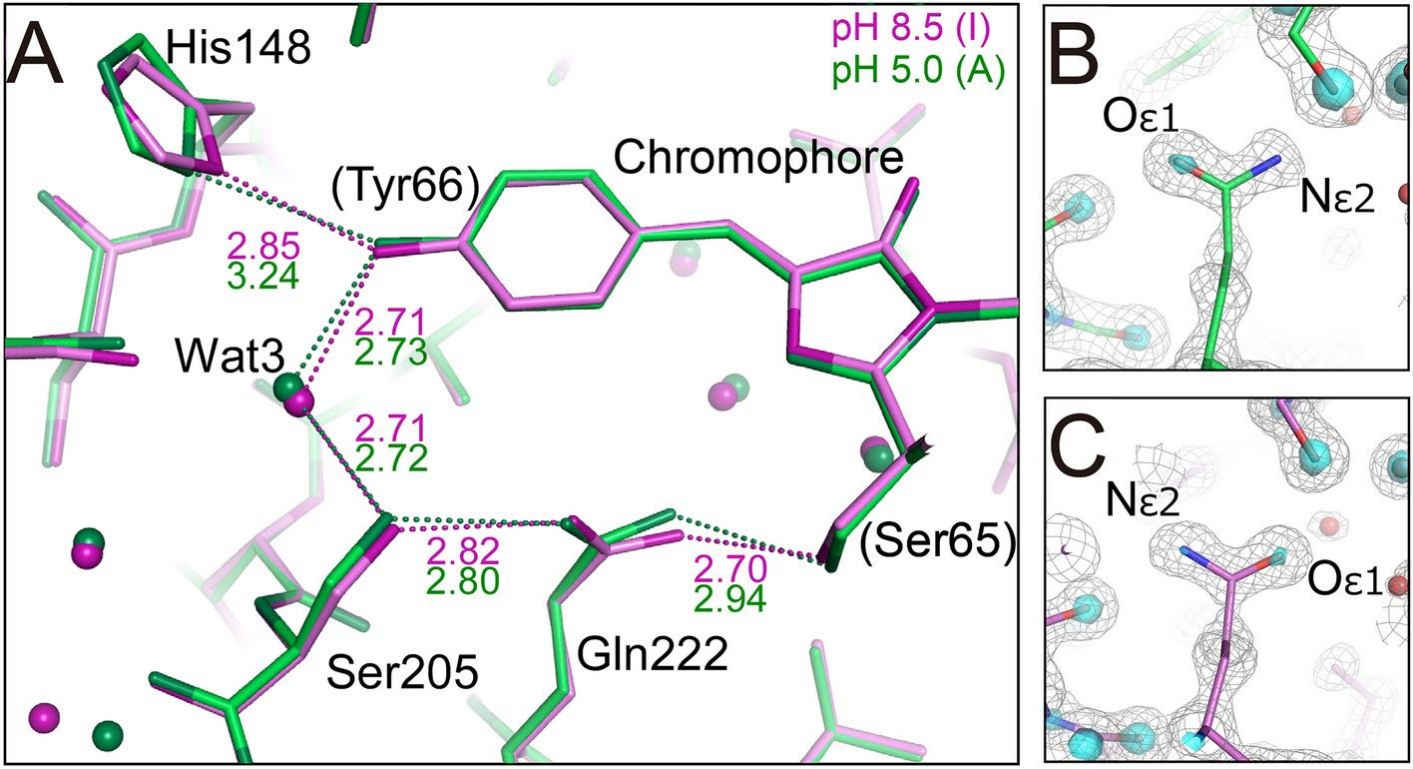
Structures of the T203V/E222Q variant at pH 8.5 and pH 5.0. (**A**) The structures at pH 8.5 (in the I state) and pH 5.0 (in the A state) are shown in green and magenta, respectively. The values in the figure are hydrogen bond distances in Å units. (**B**) The 2m*F*_obs_ – D*F*_calc_ map around Gln222 of the variant at pH 5.0 is shown as gray mesh at the 1.5σ level and cyan surface at the 4.5σ level. (**C**) The map for the variant at pH 8.5 is shown at the same contour level for comparison.

Since the T203V/E222Q variant is converted to the A state by change of pH, the crystal structure of the variant at pH 5.0 was also determined at a high resolution of 1.2 Å (Table S1, and Fig. S3A,B). A large difference from the structure at pH 8.5 is the distance between Nδ1 of His148 and Oη of the chromophore. This distance is 3.24 Å at pH 5.0, indicating weak hydrogen bonding, and 2.85 Å at pH 8.5, indicating average-strength hydrogen bonding (Fig. 4A). The conformation of the side chain of Val203 is identical at the two pHs. The Oε1 and Nε2 atoms of Gln222 are hydrogen bonded with Ser205 and Ser65, respectively, at pH 5.0 (Fig. 4B), while at pH 8.5 the hydrogen bonding partners are reversed (Fig. 4C). This indicates that conformational changes of the Gln222 side chain occur in the A ↔ I conversion by pH change of the T203V/E222Q variant.

The X-ray structures of the T203V variant and wild-type GFP were determined at 1.2 Å and 1.48 Å resolution (Table S1, and Fig. S4). The conformations of the residue at position 203 are *gauche*- for both. Gln222 in the T203V variant and Glu222 in wild-type GFP are in the same conformation as Gln222 in the T203V/E222Q variant.

### Structural comparison

**Fig. 5:**
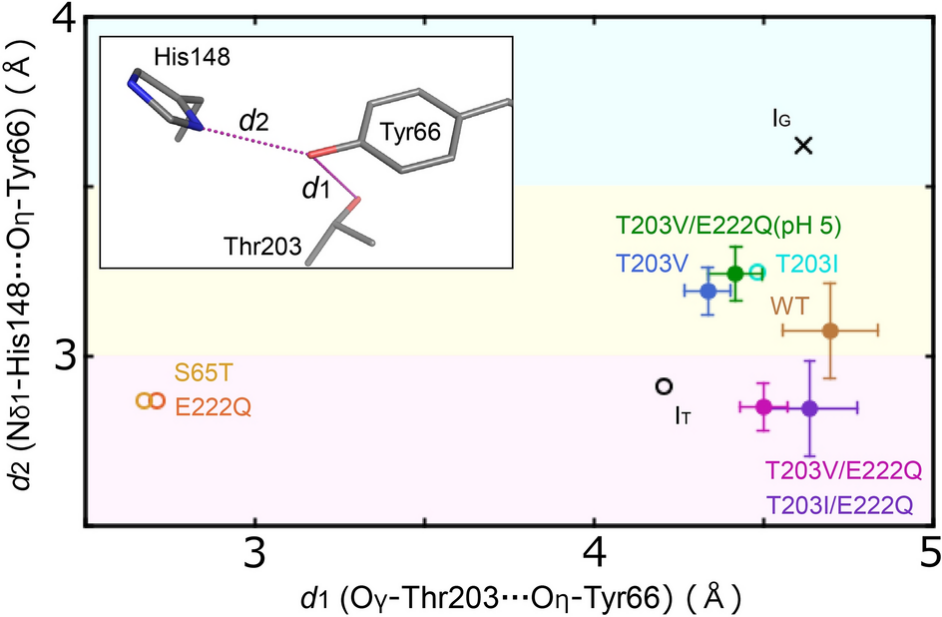
Scatter plot for the two atomic distances for structural comparison with structures in other states. The horizontal axis of the plot is the distance between Nδ1 in His148 and Oη in the chromophore, while the vertical axis is that between Oγ in Thr203 (or Cγ2 in Val or Ile) and Oη in the chromophore. The two distances are shown in the inset. Error bars derived from DPI are given to points for structures determined in this study. The points for the computational models of wild-type GFP, I_G_^14^ and I_T_ (this study), are also plotted as a black cross and a black circle, respectively. The plot areas are colored in pink, yellow or cyan according to hydrogen bond distances to give an indication of the hydrogen bond strength between Nδ1 in His148 and Oη in the chromophore: 2.5 ≤ *d* ≤ 3.0 Å (normal), 3 ≤ *d* ≤ 3.5 Å (weak), and *d* ≥ 3.5 Å (no bond).

For the distance between Nδ1 of His148 and Oη of the chromophore, average-strength hydrogen bonds were observed in the T203V/E222Q and T203I/E222Q variants. On the other hands, weaker hydrogen bonds longer that 3.0 Å were observed in the T203V variant and wild-type GFP (Fig. S4 and Fig. S5A) as well as the T203V/E222Q variant at pH 5.0. The side chain conformations of the residues at position 203 in the A-variants T203V and T203I as well as in wild-type GFP are almost identical to those in the I stabilizing variants T203V/E222Q and T203I/E222Q. This indicates that the conformation at the position 203 is the same in the A and I states. Although the distance between Nδ1 of His148 and Oη of the chromophore in the I state structure (T203V/E222Q at pH 8.5) is shorter than the structure in the A state (T203V/E222Q at pH 5.0) as stated above, the distance between Oη of the chromophore and water molecule Wat3 is almost the same (Fig. 4A). Such a trend was also observed in the comparison of other I and A structures (Fig. S5A). As for the comparison between the I and B structures, the hydrogen bonding between His148 and the chromophore exists in the structures of the S65T and E222Q variants stabilized in the B state^22^ (Fig. S5B). The side chain conformations of the residues at position 203 in the B-variants S65T and E222Q are different from those in either the I-variants or the A-variants. It is therefore clearly indicated that the state of GFP can be described by these two structural parameters—namely, the distance between Nδ1 of His148 and Oη of the chromophore, and that between Oγ of Thr203 (or Cγ2 of Val or Ile) and Oη in the chromophore. On a plot for the two structural parameters, the three states appear in different regions (Fig. 5). In other words, structures spectroscopically suggested to be in the I state (T203V/E222Q and T203I/E222Q) is positioned in the low right area in the plot, while those to be in the A (T203V/E222Q at pH 5.0, T203V, T203I and wild-type GFP) and B (S65T and E222Q) states are in the middle-high right and low left areas, respectively.

### NCI analysis of T203V/E222Q

**Fig. 6:**
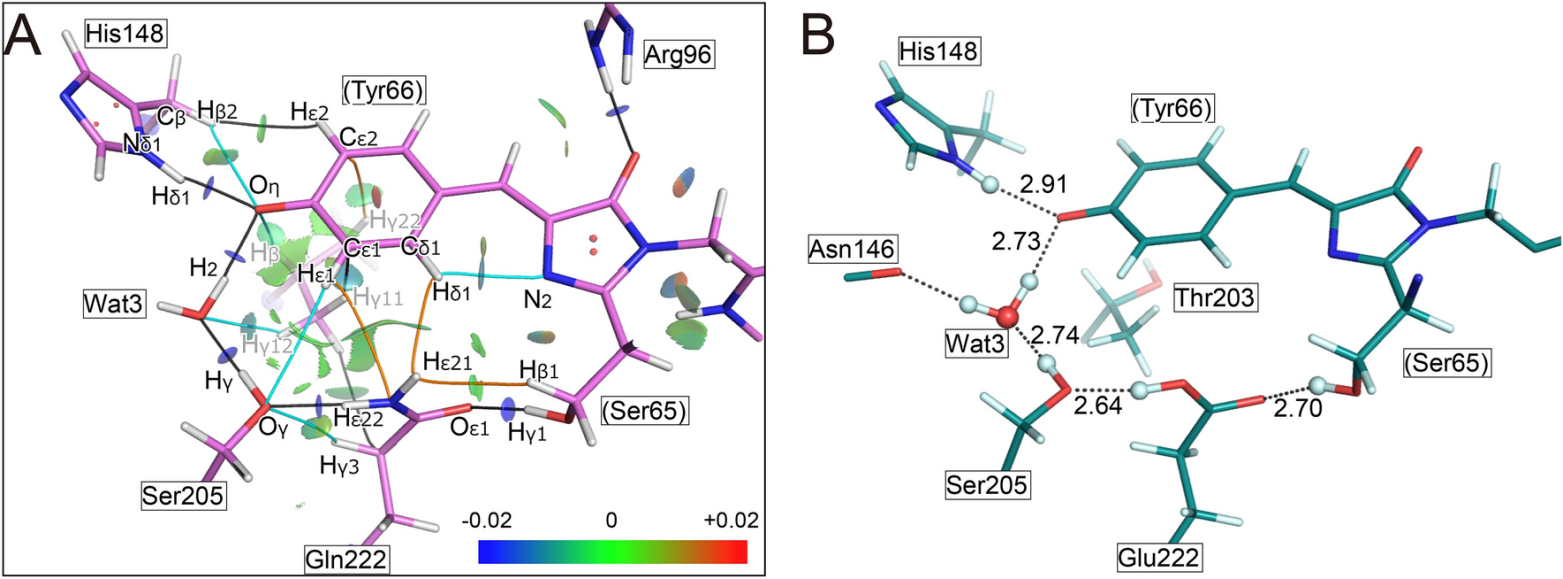
Structural properties of the I state. (**A**) The NCI plot for the T203V/E222Q variant. Hydrogen atoms are supplemented by the QM/MM optimization. The reduced density gradient isosurface at *s*(***r***) = 0.4 is represented. The color scale is given at the right side, which is determined according to the value of sign(*λ*_2_)*ρ* in an atomic unit (*e/a*_0_^3^), where *λ*_2_ is the second eigenvalue of the Hesse matrix of *ρ*. Red, green and blue correspond to repulsion, weak attraction and attraction, respectively. Bond paths for CH···O/N type hydrogen bonding are shown as cyan curves, and the paths not observed in the I structure of the wild-type GFP are shown as yellow curves. Other bond paths are shown as gray curves. (**B**) The proposed structure of wild-type GFP in the I state (I_T_). The values are hydrogen bond distances between donor and acceptor atoms of the hydrogen bonds, while the dotted lines are drawn between hydrogen and acceptor atoms.

To investigate the interaction around the chromophore in detail, the optimized structure with complemented hydrogen atoms was modelled from the crystal structure of T203V/E222Q by the QM/MM method (Fig. S6). Non-covalent interaction (NCI) plots were used to analyze the interaction, with the isosurface of the reduced density gradient calculated from the electron density ρ providing detailed information on the interaction^24^. In the NCI plot, blue surfaces indicating the presence of strong attractive interactions were actually observed between the donor and acceptor atoms of hydrogen bonds, such as between Oη of the chromophore and Hδ1 of His148 and between O of W3 and Oγ of Ser205 (Fig. 6A). In addition, the presence of CH∙∙∙O/N type hydrogen bonding interactions, as revealed by green surfaces, was observed between Hβ2 on Cβ of His148 and the Oη of the chromophore, between Hδ1 and N2 both in the chromophore, and between Hε22 of Gln222 and Oγ of Ser205. Some additional interactions formed only in the variant were observed between Hβ1 of Ser65 and Hε21 of Gln222, and between a hydrogen atom Hγ22 of the Cγ2 of Val203 and Cε2 of the chromophore. A green surface indicating the presence of CH∙∙∙N type hydrogen bonding is formed between Hδ1 of the chromophore and Nε2 of Gln222, while similar CH∙∙∙O type hydrogen bonding may be formed in wild-type GFP. Since all interactions associated with the substituted residues are weak attractive interactions, as indicated by the green surfaces, the effect on the chromophore may be slight.

### Prediction of the I-state structure in wild-type GFP

The atomic structure of wild-type GFP in the I state (I_T_) was modelled by the QM/MM method from the crystal structure of the T203V/E222Q variant at pH 8.5 (Fig. 6B). The rmsd value between the estimated I structure and the initial crystal structure was 0.11 Å for atoms in the QM region, versus 0.93 Å for all atoms in the QM and MM regions. In particular, there were only negligible differences in the flatness of the chromophore between the two. There were also no substantial differences in the distances with surrounding residues. A hydrogen atom (Hδ1) on Cδ1 of the chromophore and Oε2 of Glu222 interacts with CH∙∙∙O type hydrogen bonding, instead of the van der Waals interaction in the T203V/E222Q variant. As predicted from the NCI plot of the T203V/E222Q variant, CH∙∙∙O type hydrogen bonding is also observed between Hε1 of the chromophore and Oε2 of Glu222. The side chain of Glu222 takes the *anti*-conformation where the hydrogen atom (Hε22) on Oε2 actually forms a hydrogen bond with Oγ of Ser205. The NCI plot of our I structure shows the same features as for T203V/E222Q, with the exception of the above-described interactions associated with the substituted residues (Fig. S7).

### Comparison with other I structures

Some I structures constructed with computational methods have been reported^14,15^. These have features distinct from ours for interactions between the chromophore and the surrounding residues (Table S2). Neither His148 nor Thr203 interacts with the chromophore by hydrogen bonding in the I structure by Grigorenko et al. (I_G_)^14^, where the distances between Oη of the chromophore and proton donors in these residues are longer than 3.5 Å (Table S2). Therefore, the location of I_G_ in Fig. 5 is far from I_T_. On the other hand, it was reported that both His148 and Thr203 form hydrogen bonds with the chromophore in the I structure by Coppola et al. (I_C_), although the specific distances were not provided in their paper^15^. In any case, the distance between these residues and the chromophore can be inferred to be 2.7–3.0 Å, which would also be located in a different position in the scatter plot in this case. The conformation of Thr203 seems to be *trans* (~ 180°), which is different from the conformations in I_G_ as well from our conformation (Table S2). Ser65, which is a component of the chromophore, takes the same conformation with an χ1 value of ~ 180°. As for the orientation of Wat3, one of the acceptor atoms of hydrogen bonding is Oη of the chromophore in all three I structures. On the other hand, another hydrogen acceptor is O of Thr203 only in the I_G_, and O of Asn146 in the I_C_ and I_T_ structures.

## Discussion

**Fig. 7:**
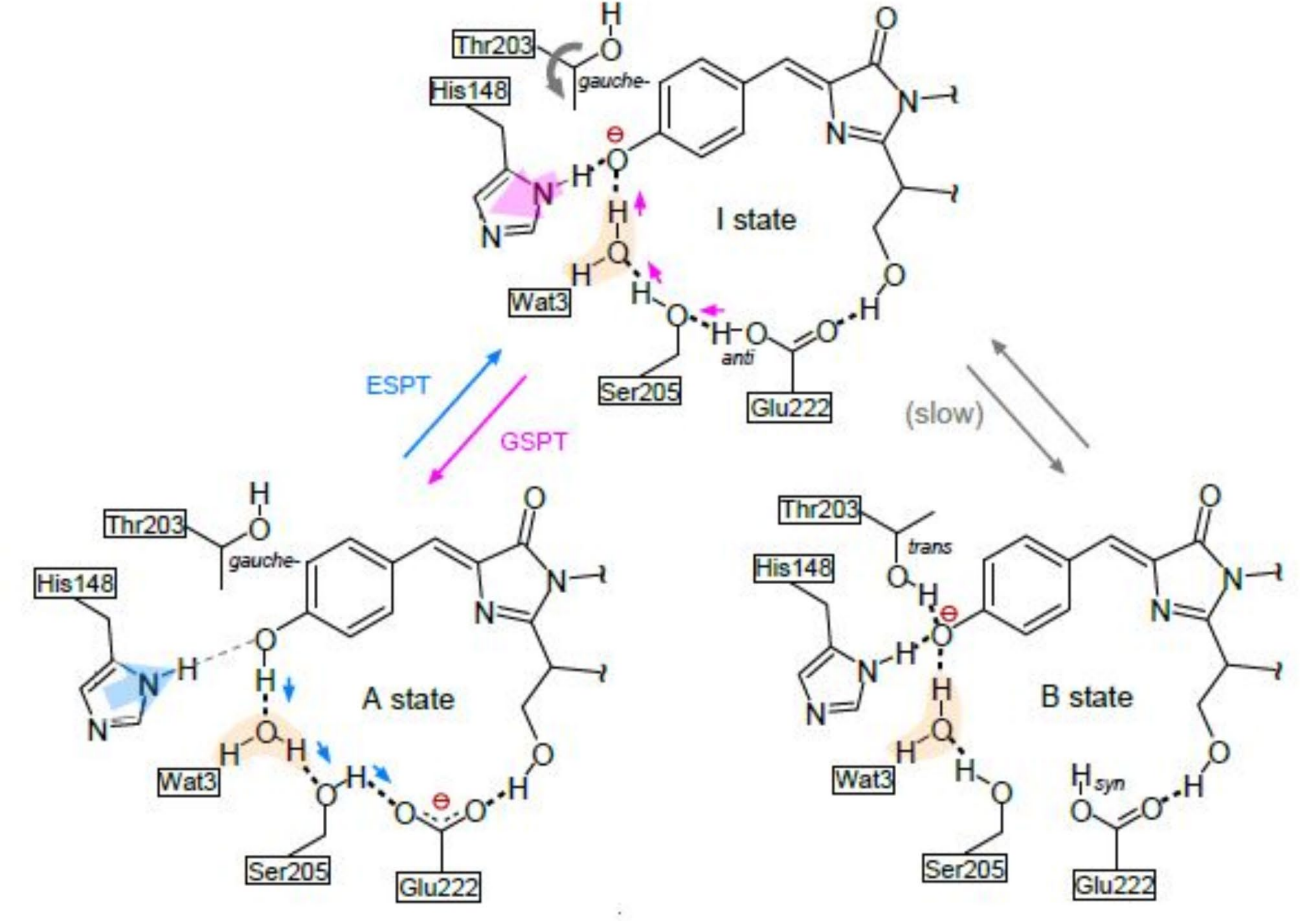
Schematic representation for the proton relay network around the chromophore in the A, B and I states. Thick and thin dotted lines represent average-length and elongated hydrogen bonds, respectively. Blue and pink arrows near hydrogen atoms indicate the proton transfer associated with the ESPT and GSPT reactions. Large blue and pink arrows on His148 indicate the movement of the side chain of the residue. Wat3 is shown in orange shading.

The atomic model of wild-type GFP in the I state constructed by the QM/MM method based on the crystal structure was differ from previously proposed structures with respect to the manner of interactions between the chromophore and the surrounding residues (Fig. 7). Thr203 is one of the mutated residues in the I stabilizing variants. In wild-type GFP, the interaction between Oγ1 of Thr203 and Oη of the chromophore stabilizes the anionic B states^25^. In the I state, almost all of the schematic and atomic models lack such an interaction; only I_C_ has it^15^. The interaction is absent in our estimated structure in addition to the crystallographic variant structures. Therefore, it is plausible that Thr203 forms no hydrogen bond with the chromophore. As for another substituted residue, Glu222, it has been thought that Glu222 is deprotonated to be the anionic form in the A state, but is protonated to be a neutral form with an *anti*-conformation in the I state. Our present analysis provides such a conformation.

No definitive conclusion has yet been reached as to the presence or absence of hydrogen bonding between His148 and the chromophore. When the schematic structure of the I-form was first proposed based on a structural comparison of the wild-type structure in the A state and the S65T structure in the B state, a hydrogen bond was assumed to be present^11^. However, there are also schematic models without the hydrogen bond^12,18,19^. The two computational atom models I_G_ and I_C_ described above differ with respect to the presence of the hydrogen bonds^14,15^. A computational study showed that Nδ1 − Hδ1 of His148 promotes proton transfer^26^. Furthermore, ab initio molecular dynamics simulations of the excited states showed the approaching His148 and chromophore just before the ESPT at the A^*^ → I^*^ transition^27^. Our I model, in which Oδ1 of His148 and Oη of the chromophore form a hydrogen bond, is consistent with the results of these studies for proton transfer.

It is strange at first appearance that the hydrogen bond between the chromophore Oη and Wat3 is not shorter than that in the A state, even though Oη is negatively charged in the I state. However, the stabilization of the negative charge on Oη is interpreted as due to the proximity of His148 to the chromophore instead of Wat3. The stabilized negative charge can facilitate the re-protonation reaction of Oη in the GSPT reaction (I → A). On the other hand, the negative charge is reported to be localized on O2 in the A* and I* states instead of Oη due to the flow of electrons from the phenolic portion to the imidazolinone portion during the electronic excitation in the A state^28^. Therefore, stabilizing the negative charge on Oη in the I state is considered to promote the I* → I transition by emission. Indeed, the fluorescence quantum yield is 0.58 for the H148V variant^29^, decreased from 0.78 for the A state of wild-type GFP^30^. The present study thus indicates that the movement of His148 is important for the efficient progress of the photoreaction cycle (A → A* → I* → I → A).

Although the structure of the I state obtained in this study is stabilized by the introduction of mutations and does not directly capture the intermediate states transiently produced during the actual photoreaction, it is sufficiently plausible as discussed above. Therefore, the present study is expected to contribute largely to the further investigations into the emission mechanism of fluorescent proteins at the atomic level.

## Materials and methods

### Preparation of samples

A pET21a-based plasmid for expression of the T203V/E222Q variant was constructed from the previously reported plasmids for expression of the E222Q variant^22^ with the inverse-PCR method. The *Escherichia coli* BL21(DE3)pLysS cells (Invitrogen, Carlsbad, CA) harboring the plasmid were grown at 37°C in LB medium supplemented with 45 mg/mL chloramphenicol and 50 mg/mL ampicillin. Induction was performed at OD_600_ ~ 0.6 by adding 1 mM IPTG, and the cultivation was further continued overnight at 22 °C. Collected cells were mixed with lysate buffer containing 50 mM Tris–HCl (pH 8.5) and Bugbuster (Novagen, Darmstadt, Germany), and shaken for ~ 24 h at room temperature. The initial step of purification was performed with an Ni–NTA affinity column (Qiagen, Germantown, MD). The protein was eluted with a solution containing 300 mM imidazole, 200 mM sodium chloride and 20 mM Tris–HCl (pH 8.5). The His-tag sequence was removed by addition of 1/100 weight of subtilisin and 200 mM ammonium sulfate to the eluted sample. The protein was further purified by anion-exchange chromatography with a Toyoperl DEAE 650S resin (Tosoh, Tokyo, Japan), followed by gel filtration with a Superdex75 column (GE Healthcare, Chicago, IL). The purified protein was dialyzed against 20 mM Tris–HCl (pH 8.5) and concentrated to ~ 15 mg/mL. The plasmid for the T203I/E222Q variant and that for the T203V variant were constructed by the same method from the plasmids of E222Q and wild-type GFP, respectively. Expressions and purifications of the T203I/E222Q and T203V variants and wild-type GFP were carried out in the same way as for the T203V/E222Q variant. All variants and wild-type GFP additionally possess cycle3 mutations (F99S/M153T/V163A) which do not affect the spectral properties or structure^20–22^.

### Spectroscopic measurements

The UV–Vis absorption spectra of the purified samples were measured with a NanoPhotometer-NP80 spectrophotometer (Implen, Munich, Germany). The GFP variants (~ 0.1 mg/mL) were dissolved in 100 mM NaCl and 20 mM Na/K phosphate (pH 7.4). For the measurement of the pH dependence, the pH values of sample solutions containing 0.1 mg/mL protein and 100 mM NaCl were set to various pH values in a range from 4.0 to 8.5 using a wide-range buffer consisting of 10 mM citrate, 10 mM MES, 10 mM MOPS, and NaOH^31^. The actual pH values of the solutions were measured with a B-712 pH meter (HORIBA, Kyoto, Japan) immediately after the measurements of the spectra. Three samples were prepared and measured for each pH value. The pK_a_ values were derived by weighted least square fitting to the Henderson–Hasselbalch equation.

### Crystallographic analysis

Crystals of T203V/E222Q were prepared in almost the same manner as those of the S65T variant^22^. First, microseed crystals of the S65T variant were prepared at 35°C with the hanging-drop vapor-diffusion method, where a sample solution containing ∼10 mg/mL protein and 20 mM Tris–HCl (pH 8.5) was mixed with an equal volume of a precipitant solution consisting of 20% (w/v) PEG4000, 25 mM MgCl_2_, and 20 mM Tris–HCl (pH 8.5). Clusters of microcrystals were crushed in the precipitant solution. Microseed crystallization was performed by the sitting-drop vapor-diffusion method at 35°C. Shortly after the making of the crystallization solution, a 0.3 µL of a seed solution consisting of a suspension of seed crystals was added to a 10 µL crystallization solution consisting of ~ 5 mg/mL protein, 8% (w/v) PEG4000, 12.5 mM MgCl_2_, and 10 mM MES-NaOH (pH 5.0). The solution was equilibrated against a 500 µL reservoir solution containing 16% (w/v) PEG4000, 25 mM MgCl_2_, and 20 mM MES-NaOH (pH 5.0). Single crystals with a length of ~ 0.5 mm were obtained within 3 days after microseeding. For the preparation of crystals of T203V/E222Q at pH 8.5, the crystals were immersed in a series of solutions with stepwise changes of pH and the concentration of PEG4000. The final solution contained 40% (w/v) PEG4000, 25 mM MgCl_2_, and 20 mM Tris–HCl (pH 8.5). However, the pH was not changed in the preparation of crystals at pH 5.0. The soaked crystals were frozen in liquid nitrogen. Crystals of the T203I/E222Q variant, T203V variant and wild-type GFP were prepared in the same way.

Diffraction data were collected at BL41XU of SPring-8 (Hyogo, Japan). Crystals were cooled at 15 K. The wavelength of X-rays was set to 0.70 Å. Diffraction spots were recorded with an Eiger X 16M detector (Dectris). The helical data-collection method was employed in order to suppress the dose for each irradiated position. The maximum dose for each position of the crystal was set to ~ 10^4^ Gy, as was the case for the T203I variant in the previous study^22^. The absorption dose was estimated with the RADDOSE program^32^.

The diffraction data were processed with the XDS program^33^. The resolution limits of data were determined to be a shell of < *I*/σ(*I*) >  ~ 2 (refs.^34,35^). The structures were solved by the molecular replacement method using a GFP structure (PDB ID: 6JGJ) as an initial model^22^. To calculate *R*_free_, the same set of 5% of all reflections was selected as a test set for all data sets. The refinement calculations of T203I/E222Q (pH 8.5) were carried out using the Phenix_refine program in the PHENIX suite^36^. The geometric restraint for the anionic chromophore was the same as for 6JGJ^22,23^. The structures were manually corrected with the COOT (v0.9.6) program^37^ according to the 2m*F*_obs_ − D*F*_calc_ and *F*_obs_ − D*F*_calc_ maps. The alternative conformations with an occupancy greater than 0.15 were included in the final models. The coordinate errors for the respective atoms (σ_i_) were estimated from σ_average_ or DPI using the formula σ_i_ = σ_average_ (*B*_i_/*B*_average_)^1/2^. The errors for the interatomic distance were derived according to σ_l_ = (σ_i_^2^ + σ_j_^2^)^1/2^. Figures for the molecular models were generated with the PyMol (v2.6.0a0) program^38^.

### QM/MM calculations

For the T203V/E222Q variant, the main conformations of all residues and 497 waters in the crystal structure were selected for the QM/MM calculations (Fig. S6A). The QM region contains Arg96, His148, Val203, Ser205, Gln222, the chromophore and Wat3 (Fig. S6B). Hydrogen atoms were complemented with the PDB2PQR (v3.5.2) program^39^, while the protonation states for dissociable residues in the crystallization solution (pH 8.5) were estimated with the PROPKA program^40^. The initial model was placed in a water box of 65 × 65 × 75 Å^3^ including 8855 waters. The energy optimized structure was obtained by the QM/MM method by using the GAMESS (US) (vSep.30.2020.R2) program suite^41^. The CHARMM36 force field were applied to the MM region^42^. The QM calculations were performed at the PBE0/6–31(d) level, which has been utilized for GFP^14^. The hydrogen atoms of water molecules were optimized with the MM calculation in the first step. The hydrogen atoms of the QM region were optimized in the next step. In the final step, all atoms of the QM region were optimized. As for the I structure of wild-type GFP, the initial structure was constructed from the crystal structure of the T203V/E222Q variant at pH 8.5 with COOT^37^. The side chains of the replaced residues were kept in the initial conformation. The energy-optimized structure was obtained in the same way as that of the T203V/E222Q variant.

The NCI analyses were performed using the DFT electron density calculated at the PBE0/6–311(d,p) level. The NCI surfaces were plotted with the NCIPLOT (v4.0) program^43^. Bond paths were detected with the Multiwfn (v3.8) program^44^.

## Supplementary Information

Supplementary Information.

## Data Availability

The crystallographic data have been deposited in the Protein Data Bank with accession codes 8ZUP (for T203V/E222Q at pH 8.5), 8ZUQ (for T203I/E222Q), 8ZUR (for T203V/E222Q at pH 5.0), 8ZUS (for T203V) and 8ZUT (for wild-type GFP).
