# Supplementary material for: Structural characterization of green fluorescent protein in the I-state
Source: Sci Rep. 2024 Oct 1;14:22832. doi: 10.1038/s41598-024-73696-y (PMC11445422; doi:10.1038/s41598-024-73696-y)
Supplement: Supplementary file 1 — Supplementary Information. [file 41598_2024_73696_MOESM1_ESM.pdf]

Supplementary Information

Structural characterization of green fluorescent protein in the I-state

Ryota Takeda, Erika Tsutsumi, Kei Okatsu, Shuya Fukai, and Kazuki Takeda*

Department of Chemistry, Graduate School of Science, Kyoto University, Sakyo-ku, Kyoto 606-8502, Japan

* To whom correspondence may be addressed.

Email: ktakeda@kuchem.kyoto-u.ac.jp

Supplementary Table S1. Data collection and refinement statistics.

	T203V/E222Q (pH 8.5)	T203I/E222Q (pH 8.5)	T203V/E222Q (pH 5.0)	T203V (pH 7.5)	wild-type (pH 8.5)
**Data collection**					
Space group	$P2_12_12_1$	$P2_12_12_1$	$P2_12_12_1$	$P2_12_12_1$	$P2_12_12_1$
Cell dimensions					
a (Å)	50.70	50.50	51.50	50.72	50.54
b (Å)	62.27	62.45	57.79	62.24	62.35
c (Å)	68.43	67.78	68.67	68.34	67.76
Resolution (Å)	50−1.20 (1.27–1.20)*	50−1.48 (1.57–1.48)*	50−1.20 (1.27–1.20)*	50−1.20 (1.27–1.20)*	50−1.48 (1.57–1.48)*
R_{sym} (%)	15.5 (55.8)	16.0 (81.3)	8.7 (79.8)	10.9 (61.3)	15.2 (98.8)
$<I/\sigma(I)>$	6.5 (2.2)	7.3 (2.0)	10.8 (2.0)	10.0 (2.1)	9.0 (2.0)
Completeness (%)	99.8 (99.2)	100.0 (100.0)	100.0 (100.0)	99.9 (99.6)	100.0 (100.0)
Redundancy	6.8	6.7	6.7	6.7	6.6
$CC_{1/2}$ (%)	99.4 (84.5)	99.4 (80.7)	99.4 (84.5)	99.9 (84.1)	99.4 (84.5)
**Refinement**					
Resolution (Å)	50−1.20	50−1.48	50−1.20	50−1.20	50−1.48
Total reflections	68,179	36,405	64,720	68,327	36,330
R_{work}/ R_{free} (%)	13.9/17.3	15.6/19.3	12.9/16.6	13.3/16.5	16.1/19.7
Coordinate error (Å)	0.070	0.128	0.066	0.069	0.122
No. of atoms					
Protein	1968	2043	2010	2037	1872
Chromophore	21	21	21	21	21
Water	600	525	479	654	427
Ion	2	2	2	2	2
B-factors (Å^2)					
Protein	10.4	14.5	13.9	11.9	16.5
Chromophore	6.6	8.7	10.0	6.6	10.0
Water	27.2	27.5	26.0	24.8	29.7
Ion	10.9	12.4	15.4	9.4	12.9
Rmsd					
Bond lengths (Å)	0.008	0.005	0.014	0.007	0.009
Bond angles (°)	1.06	0.83	1.34	0.98	1.01
PDB code	8ZUP	8ZUQ	8ZUR	8ZUS	8ZUT

*Values for the highest resolution shell are in parentheses.

Supplementary Table S2. Structural comparison with other I structures.

	I_G	I_C [a]	I_T
Methods	QM/MM	QM/MM	QM/MM
Initial structure	1EMB	1EMA	8ZUP
Distance of Oη_CRO − Nδ1_His148	3.62 Å	(H-bonding)	2.91 Å
Distance of Oη_CRO − Oγ1_Thr203	4.62 Å	(H-bonding)	4.21 Å
χ1 of Thr203	-42.4° (*gauche-*)	(*trans*)	-55.5° (*gauche-*)
χ1 of Ser65 (in CRO)	-168.5° (*trans*)	(*trans*)	-168.7° (*trans*)
H-bonding acceptor of Wat3	O_Thr203	O_Asn146	O_Asn146
H-bonding acceptor of Wat3	Oη_CRO	Oη_CRO	Oη_CRO
References	ref. 1	ref. 2	this work

[a] Atomic coordinates are not available. Distances and χ1 are qualitatively estimated from figures in the paper.

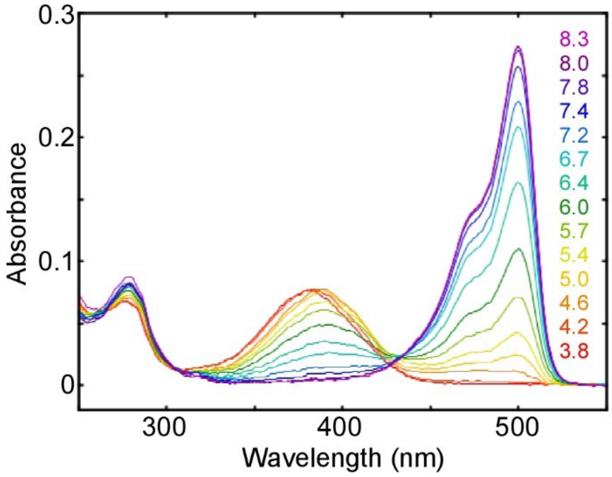

Supplementary Figure S1. UV-vis absorption spectra of the T203V/E222Q variant at various pH values are shown in a rainbow manner from low (red) to high (purple) pH. The numbers in the figure indicate the pH values.

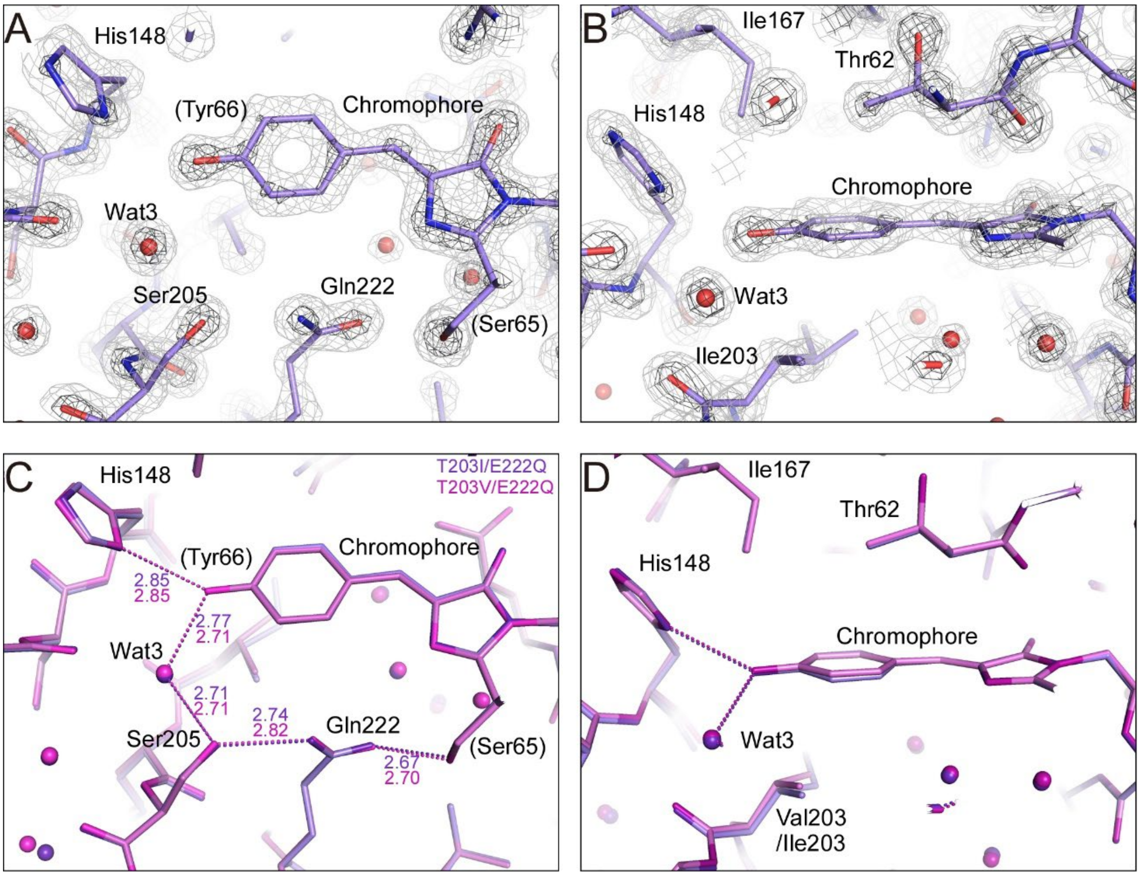

Supplementary Figure S2. Crystal structure of the T203I/E222Q variant. (**A**) The $2mF_{obs} - DF_{calc}$ map around the chromophore of the T203I/E222Q variant is shown as light gray (1.5σ) and gray (3σ) meshes. (**B**) The side view of (A). (**C**) Superimposition of crystal structures in the I state. The structure of the T203V/E222Q (pH 8.5) and T203I/E222Q are shown in magenta and violet, respectively. The values are hydrogen bond distances. (**D**) A side view of (C).

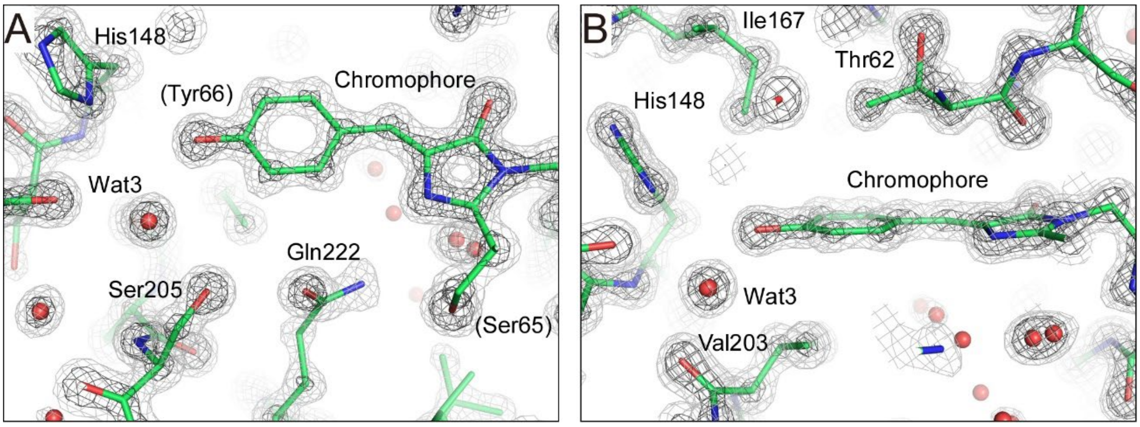

Supplementary Figure S3. Crystallographic analysis of the T203V/E222Q variant at pH 5.0. (**A**) The $2mF_{obs} - DF_{calc}$ map around the chromophore of the T203V/E222Q variant at pH 5.0 is shown as light gray and gray meshes at 1.5σ and 3σ levels. (**B**) The side view of (A).

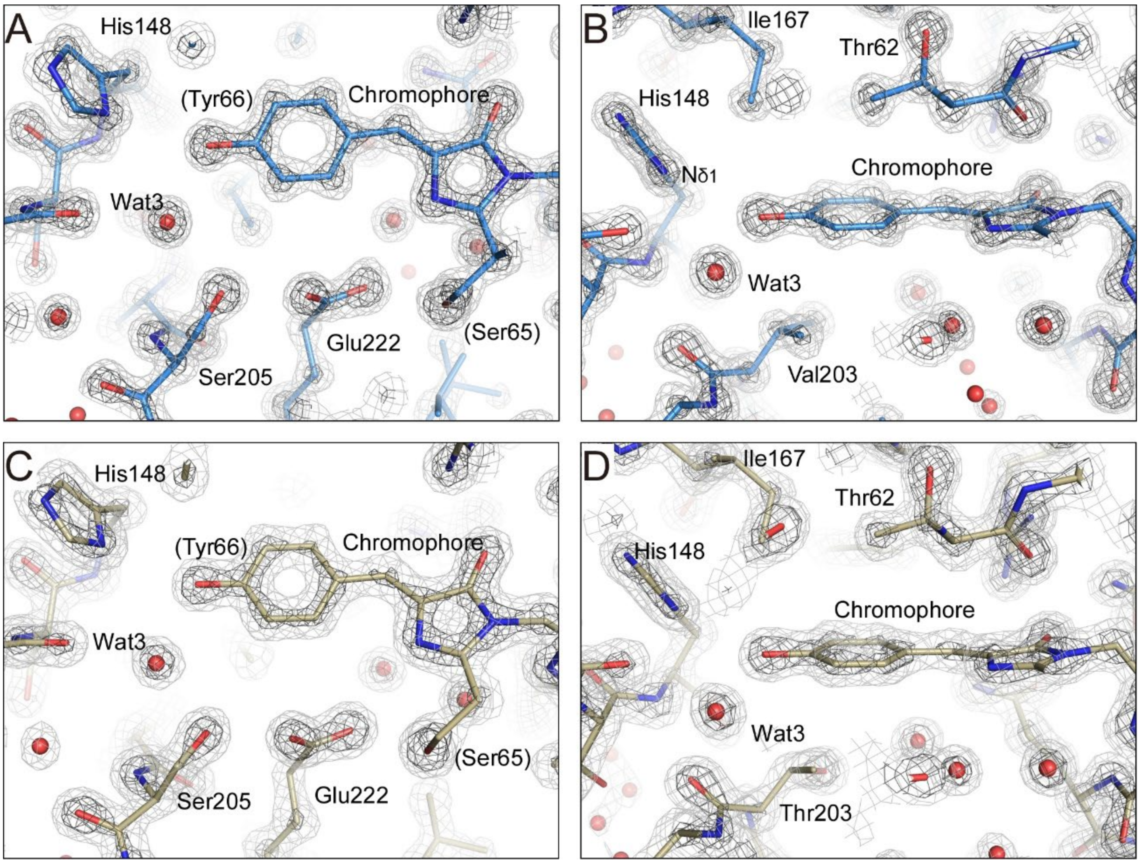

Supplementary Figure S4. Electron density maps of GFP variants in the A state. (**A**) The $2mF_{obs} - DF_{calc}$ map around the chromophore of the T203V variant is shown as light gray (1.5σ) and gray (3σ) meshes. (**B**) The side view of (A). (**C**) The map of wild-type GFP. (**D**) The side view of (C).

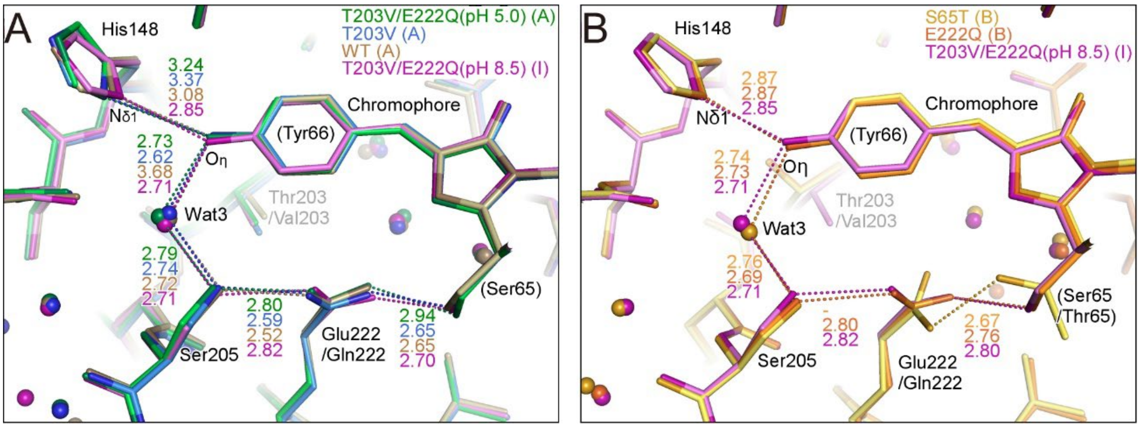

Supplementary Figure S5. Structural comparison with structures in other states. (**A**) Structural comparison between I and A forms. The structure of the T203V/E222Q (pH 5.0), T203V variants and the wild-type GFP are shown in green, blue and beige. For comparison, the structure of the T203V/E222Q (pH 8.5) in the I state is superimposed in magenta. (**B**) Structural comparison between the I and B forms. The structures of the S65T and E222Q variants are shown in yellow and orange, while that of the T203V/E222Q (pH 8.5) in the I state is superimposed in magenta for comparison.

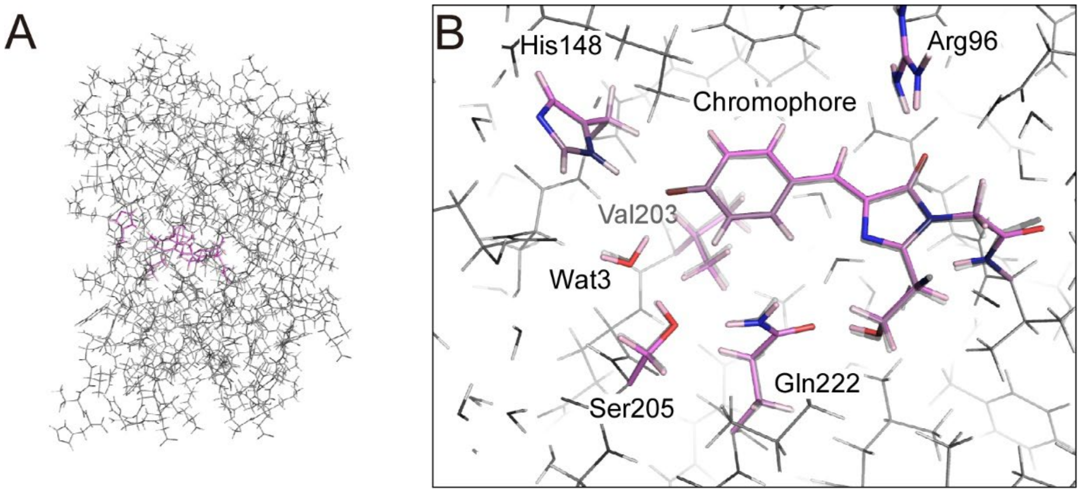

Supplementary Figure S6. QM/MM calculation of the T203V/E222Q variant. (**A**) GFP atoms in the QM region are indicated as colored sticks, while those in the MM region are shown as gray lines. Water molecules are removed from the figure, while waters in the water box of 65×65×75 $Å^3$ are included in the calculation. (**B**) A close-up view of (A) around the QM region. The QM/MM optimized (colored sticks) and the initial structures (gray lines) are superimposed.

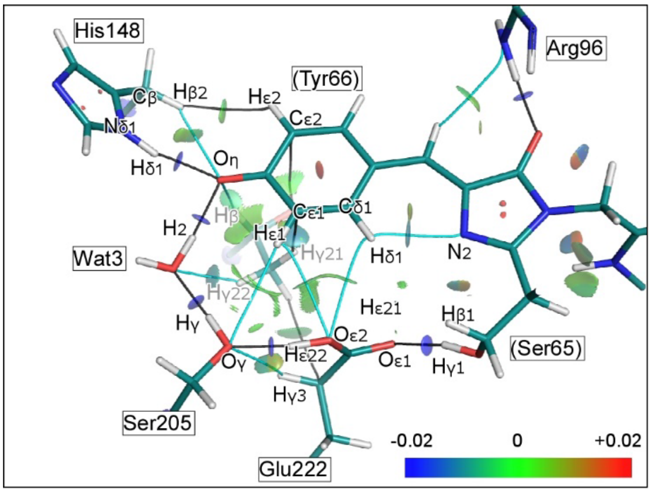

Supplementary Figure S7. The NCI plot for the proposed I state structure in wild-type GFP (I_T). The reduced density gradient isosurface at $s(\boldsymbol{r}) = 0.4$ is represented. The color scale is given at the right side, which is determined according to the value of sign(λ_2)ρ in an atomic unit (e/a_0^3), where λ_2 is the second eigenvalue of the Hesse matrix of ρ. Bond paths for CH···O/N type hydrogen bonding are shown as cyan curves, while other bond paths are shown as gray curves.

Supplementary references

1. Grigorenko, B. L., Nemukhin, A. V., Polyakov, I. V., Morozov, D. I. & Krylov, A. I. First-principles characterization of the energy landscape and optical spectra of green fluorescent protein along the A→ I→ B proton transfer route. *J. Am. Chem. Soc.* **135**, 11541–11549 (2013).
2. Coppola, F., Perrella, F., Petrone, A., Donati, G. & Rega, N. A not obvious correlation between the structure of green fluorescent protein chromophore pocket and hydrogen bond dynamics: A choreography from ab initio molecular dynamics. *Front. Mol. Biosci.* **7**, 569990 (2020).
